# *Cntnap2*-dependent molecular networks in autism spectrum disorder revealed through an integrative multi-omics analysis

**DOI:** 10.1038/s41380-022-01822-1

**Published:** 2022-10-17

**Authors:** Wooyoung Eric Jang, Ji Hwan Park, Gaeun Park, Geul Bang, Chan Hyun Na, Jin Young Kim, Kwang-Youl Kim, Kwang Pyo Kim, Chan Young Shin, Joon-Yong An, Yong-Seok Lee, Min-Sik Kim

**Affiliations:** 1 Department of Applied Chemistry, Institute of Natural Science, Global Center for Pharmaceutical Ingredient Materials, Kyung Hee University, Yongin, 17104 Republic of Korea; 2 Department of New Biology, DGIST, Daegu, 42988 Republic of Korea; 3 Department of Physiology, Department of Biomedical Sciences, Neuroscience Research Institute, Seoul National University College of Medicine, Seoul, 03080 Republic of Korea; 4 Biomedical Omic Research Group, Korea Basic Science Institute, Ochang, 28119 Republic of Korea; 5 Department of Neurology, Institute for Cell Engineering, Johns Hopkins University School of Medicine, Baltimore, MD 21205 USA; 6 Department of Clinical Pharmacology, Inha University Hospital, Incheon, 22212 Republic of Korea; 7 Department of Biomedical Science and Technology, Kyung Hee Medical Science Research Institute, Kyung Hee University, Seoul, 02447 Republic of Korea; 8 School of Medicine and Center for Neuroscience Research, Konkuk University, Seoul, 05029 Republic of Korea; 9 Department of Biosystems and Biomedical Sciences, College of Health Science, Korea University, Seoul, 02841 Republic of Korea; 10 New Biology Research Center, DGIST, Daegu, 42988 Republic of Korea; 11 Center for Cell Fate Reprogramming and Control, DGIST, Daegu, 42988 Republic of Korea

**Keywords:** Molecular biology, Neuroscience

## Abstract

Autism spectrum disorder (ASD) is a major neurodevelopmental disorder in which patients present with core symptoms of social communication impairment, restricted interest, and repetitive behaviors. Although various studies have been performed to identify ASD-related mechanisms, ASD pathology is still poorly understood. *CNTNAP2* genetic variants have been found that represent ASD genetic risk factors, and disruption of *Cntnap2* expression has been associated with ASD phenotypes in mice. In this study, we performed an integrative multi-omics analysis by combining quantitative proteometabolomic data obtained with *Cntnap2* knockout (KO) mice with multi-omics data obtained from ASD patients and forebrain organoids to elucidate *Cntnap2*-dependent molecular networks in ASD. To this end, a mass spectrometry-based proteometabolomic analysis of the medial prefrontal cortex in *Cntnap2* KO mice led to the identification of *Cntnap2*-associated molecular features, and these features were assessed in combination with multi-omics data obtained on the prefrontal cortex in ASD patients to identify bona fide ASD cellular processes. Furthermore, a reanalysis of single-cell RNA sequencing data obtained from forebrain organoids derived from patients with *CNTNAP2*-associated ASD revealed that the aforementioned identified ASD processes were mainly linked to excitatory neurons. On the basis of these data, we constructed *Cntnap2*-associated ASD network models showing mitochondrial dysfunction, axonal impairment, and synaptic activity. Our results may shed light on the *Cntnap2*-dependent molecular networks in ASD.

## Introduction

Autism spectrum disorder (ASD) is a common neurodevelopmental disorder (NDD) with rapidly increasing incidence worldwide. ASD patients present with social-psychological problems that lead to the most common symptoms: social communication impairment, repetitive behaviors, and restricted interest [1, 2]. Challenges derived from ASD affect the patients and place a burden on their families and society [3, 4]. Despite its importance, the pathobiology of ASD largely remains unknown.

Through genomic studies of large ASD patient cohorts, ample novel ASD-associated genes have been identified; these genes have been used to generate various genetically engineered mouse models with autistic-like phenotypes, and these models have contributed to a better understanding of ASD pathophysiology [5–7]. However, some mouse model experimental results are not applicable to humans due to organism differences [8, 9]. When translating mouse study findings to the clinic and interlacing results obtained from different organisms, these organism-specific differences must be carefully considered to ensure that the precise ASD etiology has been accurately recapitulated in ASD mouse models.

Genetic defects to *Contactin-associated protein-like 2* (*CNTNAP2*) can cause many neurological disabilities in humans, including ASD and intellectual disability [10, 11]. *Cntnap2* knockout (KO) mice exhibit abnormal behaviors that mimic core ASD features. Loss of *Cntnap2* led to impaired neuronal migration and reduced neuronal density in the medial prefrontal cortex (mPFC) in *Cntnap2* KO mice [12]. *Cntnap2* KO mice showed altered synaptic plasticity and imbalanced excitation/inhibition of neural networks [2]. Ample research with *Cntnap2* KO models has revealed that *Cntnap2* was associated with neuronal circuit development [13, 14]. However, the pathophysiology underlying these outcomes is still poorly understood.

Advances in high-throughput molecular profiling (omics) techniques have allowed researchers to understand a wide range of molecular features (e.g., mRNAs, proteins, and metabolites) and have revealed the molecular mechanism underlying NDDs [1, 11, 15–18]. In addition to global gene expression profiles with the complex mixture of cells comprising bulk transcriptome samples, single-cell RNA sequencing (scRNA-seq) has enabled the investigation of gene expression profiles at the individual cell level [19]. Proteins and metabolites that play important roles in ASD pathophysiology can be comprehensively profiled through mass spectrometry (MS)-based proteomic or metabolomic techniques [20–22].

We have implemented a variety of omics data obtained from the forebrains of mice and humans and from patients’ organoids to identify molecular networks in ASD. Taking advantage of the technical benefits conferred by omics research, we investigated *Cntnap2*-associated ASD molecular networks in the prefrontal cortex (PFC) [23]. We performed proteometabolomic analysis with the mPFC of *Cntnap2* KO autistic mice, and found *Cntnap2*-associated molecular features. By integrating the matched expression direction of our mouse model results with ASD patient PFC multi-omics data, we identified a set of significant ASD-related genes. By reanalyzing the scRNA-seq of forebrain organoids of ASD patients with *Cntnap2* mutations, we identified important cell types in *Cntnap2*-dependent ASD. Ultimately, through this integration of various omics data, we constructed cellular network models of mitochondrial dysfunction, axonal impairment, and synaptic activity that represent *Cntnap2*-dependent ASD networks.

## Materials and methods

### Experimental model

Male *Cntnap2*^−/−^ mice (Stock No: 017482) from The Jackson Laboratory (USA) were used for breeding and mating to produce *Cntnap2*^+/+^ (wild-type), *Cntnap2*^+/−^ (heterozygous KO), and *Cntnap2*^−/−^ (homozygous KO) mice. The detailed information about subjects and the behavior test methods are provided in Supplementary Information (Supplementary Fig. S1). Experiments were conducted in accordance with the guidelines approved by the Institutional Animal Care and Use Committee of Seoul National University (IACUC #: SNU 171220-2-5).

### Omics data generation and collection

For mouse proteomic data generation, the quantitative proteomic analysis was performed as previously described [24–26] with minor modifications. Briefly, proteins were extracted from the mPFC of *Cntnap2* KO (*n* = 5) and control mice (*n* = 5), and then enzymatically digested by trypsin. The peptides were labeled with a 10-plex TMT reagent, followed by fractionation by reversed-phase liquid chromatography (LC). Each fraction was analyzed with a high-resolution Orbitrap MS in data-dependent acquisition mode (DDA).

For mouse metabolomic data generation, the targeted metabolomic analysis was conducted according to the manufacturer’s protocols [27]. Briefly, the extracted metabolites from the mPFC of *Cntnap2* KO (*n* = 4) and control (*n* = 5) mice were spiked-in with internal standards (IS) and divided into two aliquots. The first aliquot was used to measure 21 amino acids (AAs) and 21 biogenic amines (BAs), while the second aliquot was used to analyze 40 acylcarnitines (ACs), 14 lysophosphatidylcholines (LPCs), 76 phosphatidylcholines (PCs), 15 sphingomyelins (SMs) and the hexoses. The first and second aliquots were analyzed by LC–MS/MS and flow injection analysis-MS (FIA-MS/MS) in multiple reaction monitoring mode (MRM), respectively.

In the case of human PFC omics data, the bulk RNA-seq data (including 38 healthy and 25 ASD) was downloaded from the GEO database (GSE51264 and GSE59288) [28]. The untargeted metabolomic data (including 40 healthy and 32 ASD PFC) was obtained from Supplementary Data in Kurochkin et al. [20]. The untargeted lipidomic data (including 403 healthy and 50 ASD PFC) was available at https://data.mendeley.com/datasets/m4dt3z68s5/1 [21]. For the ASD organoid data, the forebrain organoids scRNA-seq data were collected from the GEO database (GSE174569) [19]. All detailed information on the samples can be found in previous studies [19–21, 28].

A full description of this section can be found in Supplementary Information.

### Bioinformatics analysis

Differentially expressed molecular features were identified using a previously reported statistical testing method with minor modifications [29]. Briefly, adjusted p-values for the t-test (Pt), and median-ratio test (Pf) of individual molecular features for each omics data were calculated based on the permutation test (detailed methods are provided in Supplementary Information). Additionally, the Pt and Pf per metabolite or lipid from metabolome and lipidome were combined using Stouffer’s method (Pcom). For the mouse proteomic data, we considered peptides with Pt ≤ 0.05 and Pf ≤ 0.10 as differentially expressed peptides (DEPeptides). DEPeptides were summarized into differentially expressed proteins (DEPs); proteins with more than two DEPeptides were identified as DEPs, except for proteins in which the DEPeptides were found to be both up and downregulated. For the bulk RNA-seq, we considered genes with Pt ≤ 0.05 and Pf ≤ 0.10 as differentially expressed genes (DEGs). For scRNA-seq, we selected genes with Pt ≤ 0.10 and Pf ≤ 0.20 for each cell type cluster and referred to them as cell-type-specific DEGs. For the metabolome and lipidome data (mouse metabolome, human metabolome, and human lipidome), we considered metabolites or lipids with Pcom ≤ 0.05 or unique to a single group of interest as differentially expressed metabolites (DEMs) or differentially expressed lipids (DELs).

## Results

### Social behavior defects in *Cntnap2* KO mice

**Fig. 1:**
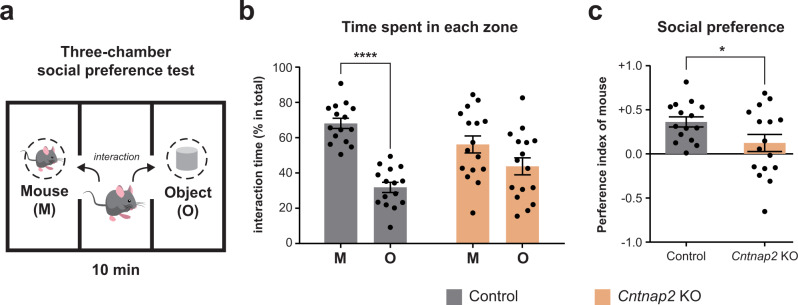
Impaired social preference in *Cntnap2* KO mice. **a** Schematic diagram used in the three-chamber social behavior tests. M: target conspecific; O: inanimate object. **b** Bar graph showing the percentage of the time spent toward a target conspecific or an inanimate object in control (*n* = 15) and *Cntnap2* KO (*n* = 16) mice. Two-way ANOVA *p* = 0.0045; Sidak’s multiple comparison test, Control: mice vs object ****p < 0.0001, *Cntnap2* KO: mice vs object *p* = 0.0634. **c** Bar graph showing preference index with significant differences between *Cntnap2* KO and control mice. Two-tailed Welch’s *t*-test *p = 0.0441.

We tested whether *Cntnap2* homozygous KO (*Cntnap2*^−/−^) mice, known as a model for ASD [10–12, 30], show a deficit in social behavior by performing a three-chamber social preference test (Fig. 1a and Supplementary Fig. S1). In the social preference test, the control littermates (*Cntnap*2^+/+^ and *Cntnap*2^+/−^) spent more time exploring a mouse than an object in the three-chamber, and the *Cntnap2* KO mice showed a comparable exploration time between the target conspecific and an object (Fig. 1b). This result shows that, compared to the control mice, the *Cntnap2* KO mice showed a significantly lower preference index toward the conspecific, confirming that *Cntnap2* gene deletion impaired the conspecific social preference of the mice (Fig. 1c). To investigate molecular changes linked to social behavior deficits, we dissected the mPFC, known as the brain area governing social preference [31], from phenotypically verified *Cntnap2* KO and control mice.

### Alteration of metabolic processes in *Cntnap2* KO mouse proteome

**Fig. 2:**
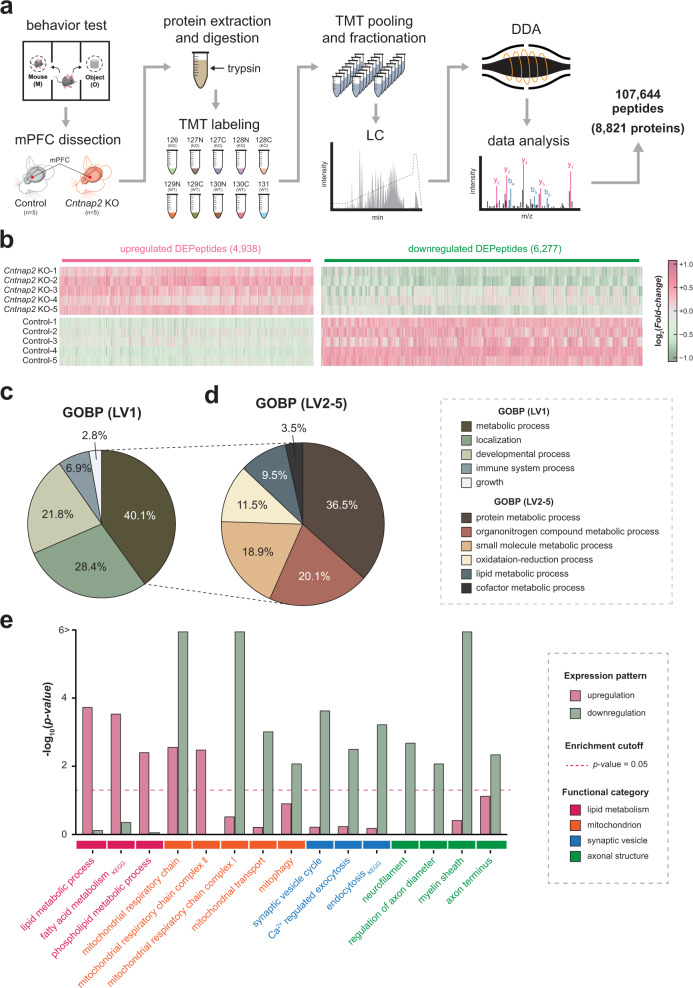
Proteomic alterations associated with *Cntnap2* KO mPFC in mice. **a** Workflow of quantitative proteomic analysis. **b** Heatmap showing the expressional difference of DEPeptides between the *Cntnap2* KO and control groups. **c**, **d** The relative proportion of DEPs according to their GOBP involvement at GOBP **c** level 1 and **d** GOBP levels 2-5. **e** Cellular processes are enriched with upregulated and downregulated proteins.

To assess the changes in protein expression in *Cntnap2* KO mice that might underlie the ASD-like phenotype, we performed a quantitative proteomic analysis with *Cntnap2* KO (*n* = 5) and control (*n* = 5) mouse mPFC tissues (Fig. 2a). A total of 8821 proteins were inferred from 107,644 nonredundant peptides in the mPFC proteome. By comparing *Cntnap2* KO and control samples, we identified a total of 844 DEPs (378 upregulated and 466 downregulated proteins) from 11,215 DEPeptides (4938 upregulated and 6277 downregulated peptides) (Fig. 2b and Supplementary Table S1–2). To explore the systematic biological processes altered by *Cntnap2* KO, a hierarchical GO analysis of the DEPs was conducted (Supplementary Table S2). Among the DEPs, the strongest GO association involved metabolic processes (40.1%) of the observed 5 comprehensive cellular categories in the GOBP (level 1) analysis, suggesting that *Cntnap2* highly affected the metabolism of the mPFC (Fig. 2c). The DEPs involved in metabolic processes were related to the metabolic processes of proteins (36.5%), organonitrogen compounds (20.1%), small molecules (18.9%), oxidation-reduction (11.5%), and lipids (9.5%) (Fig. 2d). In addition, distinctive functional characteristics were found between upregulated and downregulated DEPs (Fig. 2e and Supplementary Table S3). The upregulated DEPs were mainly involved in cellular processes related to lipid metabolisms, such as fatty acid and phospholipid metabolism, while the downregulated DEPs were closely associated with synaptic vesicle (SV) transport (synaptic vesicle cycle, Ca^2+^-regulated exocytosis, and endocytosis) and axonal compartment (neurofilament, myelin sheath, axon terminus, and regulation of axon diameter). Interestingly, upregulated and downregulated DEP was significantly associated with oxidative phosphorylation (OXPHOS), implying complicated *Cntnap2*-associated regulation of OXPHOS.

### Metabolomic profiling of *Cntnap2* KO mice

**Fig. 3:**
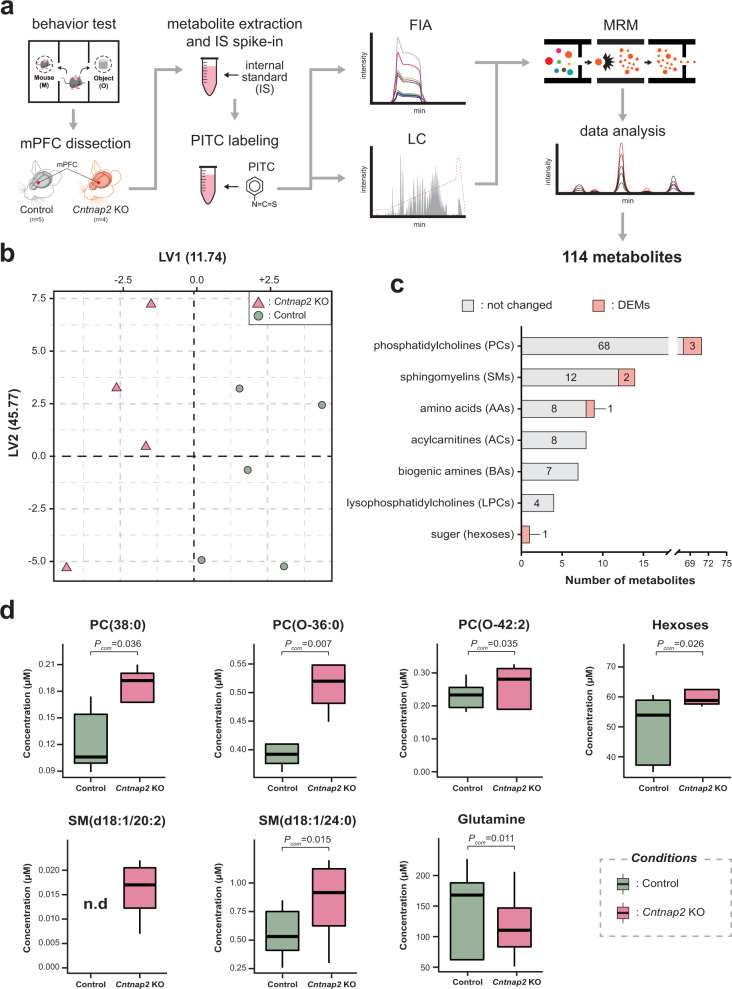
Metabolomic alterations associated with *Cntnap2* KO mPFC in mice. **a** Workflow of targeted quantitative metabolomic analysis. **b** PLS-DA plot showing the discrimination between *Cntnap2* KO from the control group. **c** The bar graph shows the number of measured metabolites in each metabolite subclass with unchanged metabolites and DEMs in each stacked bar, respectively. (d) Box plot showing DEM concentrations between *Cntnap2* KO and control groups.

As metabolism was among the top-ranked processes in the hierarchical GO analysis of proteomic results (Fig. 2c), we carried out targeted quantitative metabolomic analysis with *Cntnap2* KO (*n* = 4) and control (*n* = 5) mouse mPFC tissues (Fig. 3a). Among the 188 targeted metabolites, 114 metabolites were quantified in at least one of the samples (Supplementary Table S4). To test whether *Cntnap2* KO affected any discriminative metabolic activity, we performed a partial least squares-discriminant analysis (PLS-DA). Results showed that the *Cntnap2* KO and control groups were separated by 1st component (LV1, 11.74%), confirming altered metabolism in the *Cntnap2* KO mPFC (Fig. 3b). By comparing the metabolite concentrations, we found that 7 of 114 metabolites were DEMs (Fig. 3c and Supplementary Table S4). Specifically, glutamine (Gln), 3 phosphatidylcholines (PC; PC(38:0), PC(O-36:0), and PC(O-42:2)), 2 sphingomyelins (SM; SM(d18:1/24:0) and SM(d18:1/20:2)), and hexose were found to be significantly altered. Gln was reduced and all other DEMs were increased in the *Cntnap2* KO group (Fig. 3d). Relatively few DEMs were identified in this study (~6% of detected metabolites) compared to the number of DEPs (~12% of total proteins), leading to difficulty in proteometabolomic data integration. Because SM(d18:1/24:0) and PC(O-36:0) are among potential biomarkers in neurological disorders [32, 33], we wondered whether our proteometabolomic data represent underlying ASD mechanisms.

### Molecular characteristics shared by *Cntnap2* KO mice and ASD patients

**Fig. 4:**
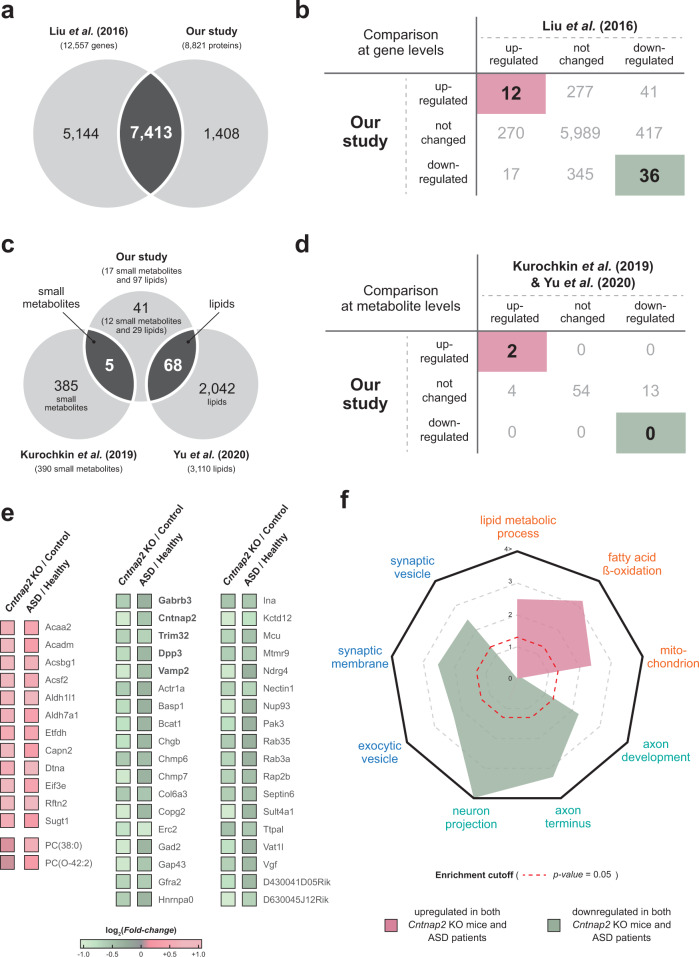
Molecular overlapping between *Cntnap2* KO mice and ASD patients. **a** Venn diagram showing the number of comparable genes in our *Cntnap2* KO mPFC proteome and the public human PFC transcriptome. **b** Pivot table showing the number of genes with corresponding expression patterns of our proteome and PFC transcriptome of ASD patients. **c** Venn diagram showing the number of comparable metabolites in our *Cntnap2* KO mPFC metabolome and the public human PFC lipidome/metabolome. **d** Pivot table showing the number of metabolites with corresponding expression patterns of our metabolome and ASD patients PFC lipidome/metabolome. **e** Heatmaps shows the expression differences of genes or metabolites in mouse and human ASD subjects compared to their corresponding controls. The genes reported as ASD-linked genes in SFARI are labeled in bold. **f** Radar plot shows cellular processes enriched with upregulated and downregulated genes in mouse and human ASD subjects.

To identify the significance of our findings, it was essential to ensure that the altered protein and metabolite levels found in mice are also found in human samples. Therefore, we accessed large publicly available human PFC datasets, including ASD cohorts. Three different datasets were used for this analysis: transcriptome from Liu et al. [28], lipidome from Yu et al. [21], and metabolome from Kurochkin et al. [20]. A total of 12,557 genes identified by Liu et al. were compared with 8821 proteins in our proteomic data (Supplementary Table S5). Of the 7413 genes found to be common to both datasets (Fig. 4a), 48 genes (12 upregulated and 36 downregulated DEGs) showed the same trend as the DEPs in our proteomic data (Fig. 4b). When we compared 114 metabolites (17 small metabolites and 97 lipids) in our metabolomic data with identified small metabolites and lipids identified by Yu et al. and Kurochkin et al., 73 metabolites (five small metabolites and 68 lipids) were detected in both datasets (Fig. 4c). Among these overlapping metabolites, PC(38:0) and PC(O-42:2) were DEMs with levels significantly increased in both *Cntnap2* KO mice and ASD patients (Fig. 4d-e, and Supplementary Table S6–8). In the case of 48 genes correlating to both *Cntnap2* KO mice and ASD patients, 36 downregulated genes were significantly associated with SV function (synaptic vesicle, synaptic membrane, and secretory vesicle) and neuronal axon (axon development, axon terminus, and neuron projection) (Fig. 4f, green, and Supplementary Table S9); these functions are pivotal for maintaining synaptic function and neuronal migration, and their dysregulation can lead to synaptic dysfunction [34] and neuronal migration deficits [35], which have been linked to NDDs [35], such as ASD and intellectual disabilities. Additionally, five downregulated genes (*Gabrb3, Cntnap2, Trim32, Dpp3*, and *Vamp2*) are genetically associated with ASD, as shown through the SFARI database [36] (Fig. 4e, bold). In contrast, 12 upregulated genes were found to be mainly involved in mitochondrial lipid metabolism (with mitochondrion, lipid metabolic process, and fatty acid β-oxidation) (Fig. 4f, pink). Thus, the proteometabolomic analysis of the mPFC in the mouse model may be a useful platform for comprehensively exploring molecular alterations.

### The excitatory neuron is a key cell type in *CNTNAP2*-dependent ASD

**Fig. 5:**
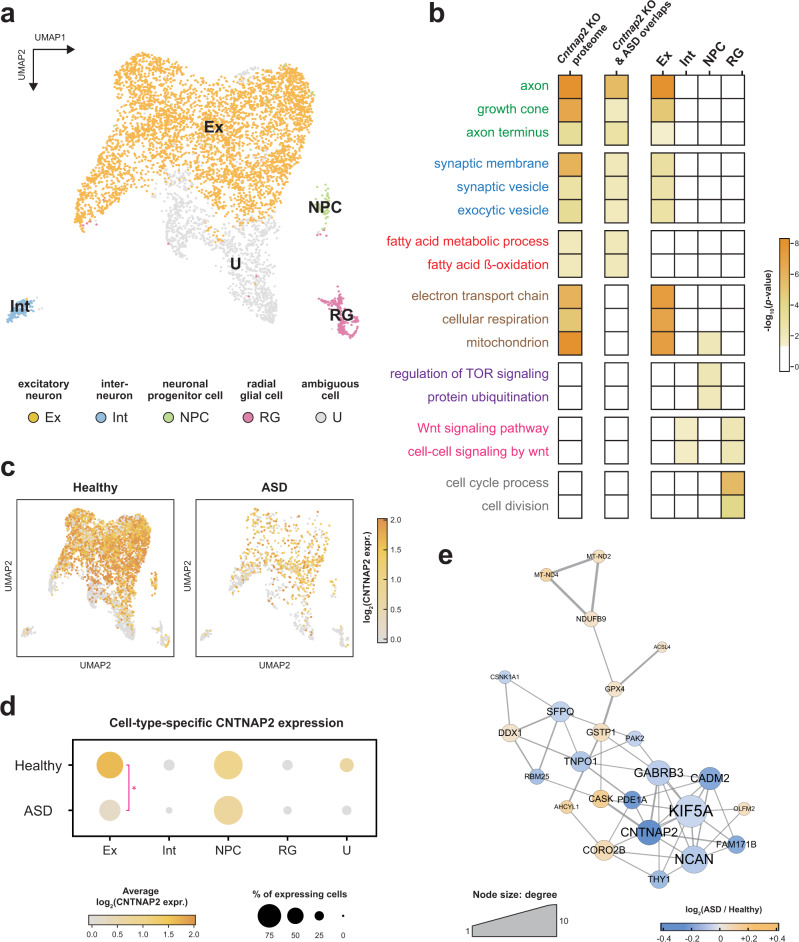
*CNTNAP2*-dependent cell types and the specific processes in which they are involved in *CNTNAP2*-deficient ASD patient-derived brain organoids. **a** UMAP visualization of the 5 representative cell types identified in the forebrain organoid DPal cells. See color legend for the cell type assignment. **b** Heatmap showing the significance of cellular processes enriched with DEPs, DEGs, and cell-type-specific DEGs identified in the mouse proteome results, ASD patient integration results, and ASD organoid results. **c** Expression of *CNTNAP2* in ASD and healthy organoid DPal cells. **d** Dot plot showing cell-type-specific *CNTNAP2* expression. The dot size and color represent the proportion of *CNTNAP2*-expressing cells and average *CNTNAP2* expression per cell-type cluster, respectively. **e** Functional protein association network of genes shown in both *Cntnap2* KO DEP and Ex-specific DEG. The node color represents the expressional difference between ASD and healthy organoids in Ex cells. The edge thickness represents the STRING interaction score.

Since the *Cntnap2* gene was deleted in the *Cntnap2* KO model mice and its expression is downregulated in ASD patients, we were interested in mapping specific networks to cell types directly affected by *Cntnap2*. To identify *Cntnap2*-affected cell types, we reanalyzed public scRNA-seq data obtained from forebrain organoids derived from ASD patients with *CNTNAP2* mutation [19]. To select PFC-specific cell types among all the cell populations in the organoids, we first performed spatiotemporal mapping analysis by comparing the in situ hybridization (ISH) data of tissues in seven different developmental stages in the Allen Developing Mouse Brain Atlas [37] using VoxHunt [38]. The best Pearson correlation was observed when ISH data from embryonic mouse day 15.5 (P15) were assessed; 5932 cells of 9392 pallium-specific cells among a total of 28,108 cells in the organoids were mapped to the dorsal pallium (DPal) (Supplementary Fig. S2a–S2c). Since DPal develops into the PFC, these mapped 5932 cells were further analyzed. The Leiden clustering method was used and led to the identification of 13 clusters, of which 5 representative cell types were manually curated using neocortex markers [39]. These cell type clusters comprised the excitatory neuron cluster (Ex; 4303 cells), interneuron cluster (Int; 153 cells), radial glial cell cluster (RG; 217 cells), neuronal progenitor cell cluster (NPC; 90 cells), and ambiguous cell cluster (U; 1169 cells) (Fig. 5a and Supplementary Table S10). Canonical cell type marker expression confirmed these cell-type assignments (Supplementary Fig. S2d).

To understand ASD-associated changes at the cell-type level, we performed DEG analysis by comparing each cell type cluster in the ASD organoids with that in the healthy normal organoids and identified a total of 1177 cell-type-specific DEGs (Supplementary Table S11): 619 Ex-specific DEGs, 121 Int-specific DEGs, 336 NPC-specific DEGs, 256 RG-specific DEGs, and 17 U-specific DEGs. With 1177 cell-type-specific DEGs, we conducted a functional enrichment analysis (Supplementary Table S12) and found that both the Int- and RG-specific DEGs were closely associated with the WNT pathway (Wnt signaling pathway, and cell–cell signaling by wnt) (Fig. 5b, pink). Only the RG-specific DEGs were enriched in the cell cycle (cell cycle process and cell division) (Fig. 5b, gray), and the TOR signal pathway (regulation of TOR signaling), while protein ubiquitination was uniquely enriched with NPC-specific DEGs (Fig. 5b, purple). When searching for the major biological processes in ASD, we compared our proteomic and ASD patient integration results and found that Ex-specific DEGs were mainly involved in axonal structure (growth cone and axon terminus), SV function (synaptic membrane, synaptic vesicle, and exocytic vesicle), and OXPHOS (electron transport chain, cellular respiration, and mitochondria), which were previously shown in this study to be pivotal processes in *Cntnap2* KO mPFC and ASD patient PFC (Fig. 5b, green, blue, and brown). Although Ex-specific DEGs did not show the association to the lipid metabolism that was found to be prevalent in *Cntnap2* KO and ASD patients (Fig. 5b, red), the Ex cluster showed the most significant functional similarity in the cell types to *Cntnap2* KO and ASD patients. Interestingly, *CNTNAP2* was generally expressed only in the Ex and NPC clusters (Fig. 5c, d). However, *CNTNAP2* was differentially expressed between the ASD and control samples only in the Ex cluster, not in the NPC cluster (Fig. 5d), suggesting that *CNTNAP2* in excitatory neurons may contribute to ASD development. When looking at Ex-specific DEGs, 24 genes showed expression patterns similar to that found in the *Cntnap2* KO proteome (Supplementary Table S11). To elucidate the *Cntnap2*-dependent molecular changes in the Ex cluster, network analysis was carried out in STRING-DB with the 24 genes. Interestingly, all 24 genes were constructed to a single connected component, and CNTNAP2 showed high hubness within a network (*p* value = 0.0767), indicating a strong association with *CNTNAP2* (Fig. 5e).

### Molecular network models associated with *Cntnap2*

**Fig. 6:**
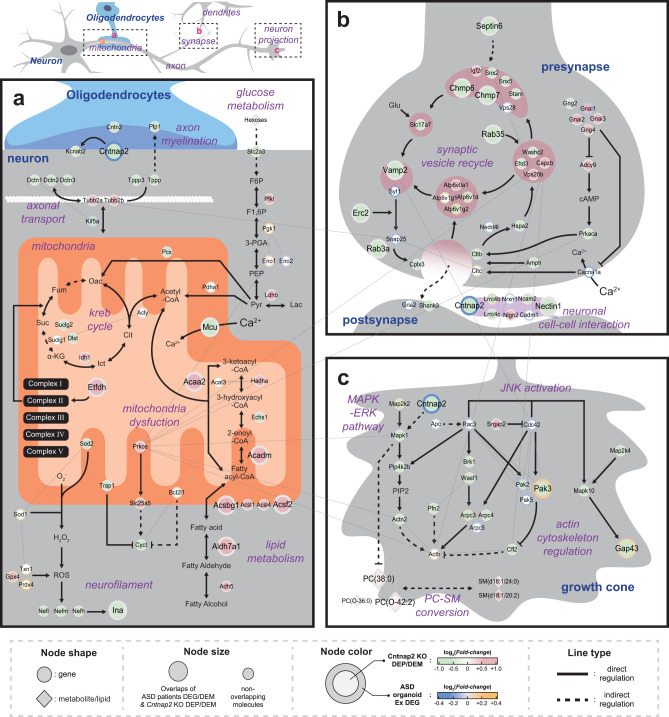
*Cntnap2*-associated ASD molecular network models. Molecular network models describing alterations in **a** myelin sheath and mitochondrial network, **b** synapses, and **c** neuron projections in the *CNTNAP2*-associated ASD PFC. Node shapes represent types of molecules. The molecules showing a significant expressional difference in mouse and human ASD subjects are displayed as larger nodes. The center and boundary colors of a node represent the expressional difference in the ASD mouse and ASD organoid Ex cells compared to their corresponding controls, respectively. Line types indicate types of regulation. Thin gray lines represent the curated protein-protein interaction.

We further investigated *Cntnap2*-associated molecular features. We first collected subsets of DEPs, DEMs, and DEGs obtained by *Cntnap2* KO mouse mPFC proteometabolomic analysis and *Cntnap2* defect organoid single-cell profiling (Supplementary Table S13). A total of 122 DEPs and 6 DEMs enriched in processes related to lipid metabolism, mitochondria, synaptic vesicle, and axonal structure were selected from our mouse data (Fig. 2d). From the scRNA-seq results, only 71 Ex-specific DEGs were involved in synaptic function, neuron projection, and mitochondria (Fig. 5d), and they were assessed to determine the aforementioned relationships between *Cntnap2* and excitatory neurons. Next, to understand ASD molecular mechanisms linked to *Cntnap2*, we generated hypothetical molecular network models with selected molecules representing molecular alterations in (1) myelin sheath and mitochondria, (2) synapse, and (3) neuron projection (Fig. 6).

The network model related to myelin sheath and mitochondria (Fig. 6a) showed systematic changes in axonal functionality and mitochondria (glycolytic process, lipid metabolism, and mitochondrial energy production). We found coordinated attenuation of axonal functional modules, including axon myelination (Cntn2, Plp1, Kcnab2, and Cntnap2), axon organization (Nefh/l/m and Ina), and axon transportation (Dctn1/2/3, Kif5a, Tppp, and Tppp3) at the protein level. In contrast, mitochondrial lipid metabolic proteins (Adh5, Aldh7a1, Acsbg1, Acsf2, Acsl1/4, Acadm, Hadha, and Acaa2) were induced by *Cntnap2* abolition in the PFC. Compared to the clear upregulated DEPs association in lipid metabolism, DEPs linked to glycolysis, and TCA cycle were unsynchronized (upregulated: Idh1, Pfkl, and Ldhb; downregulated: Suclg2, Dlst, Pcx, and Slc2a3). In the electron transport chain (ETC), most of the ETC components in DEPs, except complexes II and IV, were decreased (Supplementary Fig. S3a). At the single-cell RNA level, all mitochondria-located Ex-specific DEGs related to oxidation-reduction processes (Sod1 and Bcl2l1) and glucose metabolism (Pgk1, Eno1, Pdha1, Acyl, and Suclg1), and ETC components (Supplementary Fig. S3a) showed higher expression, suggesting higher mitochondrial contents in *Cntnap2*-deficient excitatory neurons.

The network model related to synapses (Fig. 6b) showed reduced synaptic function processes such as SV recycling and neuronal cell–cell interactions. Several major stages characterize the SV cycle, including endosome processing, SV preprocessing and docking, exo- and endocytosis, and vesicle recycling [40]. Although some SV cycle proteins in DEPs (Igf2r, Atp5v0a1, Capzb, and Vps26b) were upregulated, the majority of proteins involved in SV cycle stages were reduced: endosome processing (Septin6, Snx2/5, Stam, and Chmp6/7), SV preprocessing (Slc17a7 and Vamp2) and docking (Erc2 and Rab3a), exo-/endocytosis (Amph, Rab35, Cplx3, Cltb, and Hspa2), and vesicle recycling (Atp6v1d/g2, Washc2, and Ehd3). Similarly, Syt1 and Snap25, key SV docking elements, were downregulated in Ex-specific DEGs. Additionally, V-type ATPases (Atp6v1d/g1/g2), acidifying vesicle in the recycling stage, were upregulated in Ex-specific DEGs. SV release can be activated by cAMP-mediated signals or intercellular Ca^2+^ [41] (Fig. 6b, right). A cAMP signaling molecule (Prkaca) was reduced, whereas cAMP signal inhibiting molecules (Gnai1/2/3 and Gng4) were increased in *Cntnap2* KO mice at the protein level. At the single-cell RNA level, intercellular Ca^2+^ uptake (Cacna1a) was decreased in *Cntnap2*-deficient excitatory neurons. Neuron-contacting molecules play pivotal roles in organizing neuronal circuits and enabling synaptic connectivity in the brain [42]. We found 5 cell–cell interaction proteins among the DEPs (Cntnap2, Lrrc4b/c, Nlgn2, Ncam2, and Nectin1) and 3 cell–cell interaction-related genes (Lrrc4b, Nrxn1, and Cadm1) among the Ex-specific DEGs (Fig. 6b, bottom).

Finally, the network model related to neuron projection (Fig. 6c) showed the alteration of projection molecules and related processes. Neuronal projection is coordinated by several regulatory pathways involved in actin cytoskeleton organization, including the MAPK/ERK, RAC/CDC42/PAK, and Rho-ROCK pathways [43, 44]. The proteins related to actin cytoskeleton organization (Actn2, Pfn2, Arpc3/4, and Cfl2) and its regulatory pathway (MAPK/ERK pathway: Map2k2, Mapk1, and Pip4k2b; RAC/CDC42/PAK pathway: Brk1, Wasf1, and Pak2/3) were decreased. Rac and Cdc42 can activate the JNK pathway [45], which is a key regulatory system in neuronal migration [46]. Cntnap2 is known to regulate the MAPK/ERK pathway via IP3R1, thereby influencing neuronal migration or projection [47]. In our models (Fig. 6c, right), the protein related to the JNK pathway (Map2k4 and Mapk10) and neuronal migration (Gap43) was downregulated, which correlated with the attenuation of RAC/CDC42/PAK pathway. Although the core molecules of RAC/CDC42/PAK pathway (Apc, Rac3, Cdc42, and Pak2/5) were downregulated in *Cntnap2*-deficient excitatory neurons, some of the related molecules (Map2k2, Pak3, Arpc3, Gap43, and Cntnap2) were upregulated and showed negative expressional correlation to the *Cntnap2* KO mouse proteome. In the case of selected DEMs, increased PCs (PC(38:0), PC(O-36:0), and PC(O-42:2)) and SMs (SM(d18:1/24:0) and SM(d18:1/20:2)) can be explained by PC-SM conversion processes [48], but no enzyme involved in the conversion processes was found among the DEPs. The potential mechanism of upregulated lipids’ is the deactivation of the MAPK/ERK pathway, which led to enhanced PC biosynthesis; a previous study showed that inhibition of Erk suppressed PC biosynthesis and reduced cellular PC levels [49] (Fig. 6c, bottom).

## Discussion

Genetic alterations on the *CNTNAP2* gene, such as copy number variations, genomic inversion, single nucleotide polymorphisms, and complete loss of *CNTNAP2* gene are known to be associated with several neurological disorders. (Supplementary Fig. S4a) [12, 50–52]. To find if *Cntnap2* KO can be better explained by ASD than other neurological disorders, we carried out disease association analysis by using publicly available patients’ data from different neurological diseases (ASD and schizophrenia). We found *Cntnap2* showing strong involvement in ASD pathophysiology (Supplementary Fig. S4b). To build a molecular network of *Cntnap2*-dependent ASD at the cell type level, we implemented MS-based proteometabolomic analyses of *Cntnap2* KO mice and combined the results with the omics data obtained from human PFC and organoids with *Cntnap2* mutations. As shown in Fig. 6, the pathways involved in autism were related to the dysregulation of mitochondria, synaptic vesicle transport, and neuron projection. Here, we explored genes that were found to be interlaced between species to better describe ASD pathophysiology.

### Mitochondrial dysregulation

Mitochondrial dysfunction is one of the common molecular characteristics in ASD [53, 54]. We found that some of the mitochondrial proteins such as Mcu, Etfdh, Acaa2, Acadm, Acsbg1, Aldh7a1, and Acsf2 were altered in the mouse and human ASD subjects (Fig. 6a). MCU is essential for mitochondrial calcium uptake into the inner matrix, and plays a role in maintaining the mitochondrial membrane potential (MMP) [55]. Since Mcu was downregulated, we expect MMP discordance to disrupt mitochondrial functions. ETFDH guides several flavin-containing dehydrogenases on OXPHOS complex II and affects the activity of mitochondrial lipid oxidation and ETC system [56]. As Etfdh and OXPHOS complex II (Supplementary Fig. S3a) were upregulated, the mitochondrial lipid metabolism and ETC activity may increase. Consistently, we found that lipid metabolic enzymes such as Aldh7a1, Ascbg1, Acsf2, Acadm, and Acaa2 were increased in both the mouse and human ASD subjects. Interestingly, mutations in *ALDH7A1* have been found in NDD patients with typical symptoms of ASD [57].

### Disruption of synaptic vesicle transport

Synapse disturbance is among the most recognized molecular disruptions in various NDDs, including ASD [57]. Certain DEPs (Vamp2, Cntnap2, Erc2, Gad2, Nectin1, Rrab3a, Rab35, Chmp6, Chmp7, and Sept6) were downregulated in both the mouse and human ASD subjects, and found to be involved in SV functions in excitatory neurons (Fig. 6b). Downregulation of Rab3a, a core SV docking molecule [58, 59], indicates that vesicle transport in the SV docking was affected in ASD. Interestingly, RAB family genes have been identified as ASD-associated genes [60], and downregulation of Rab3a has been shown in other ASD models [61]. VAMP2 affects SV docking to syntaxin and SNAP25, and downregulated Snap25 in Ex clusters of ASD organoid indicated a reduction in binding efficiency. Mutations in Vamp2 have been implicated in vesicle fusion impairment in ASD [62]. Furthermore, Rab35 plays a role in endocytic recycling; specifically, a SV-reformation is transported from the early endosome to replenish the SV pool [63]. Mutations in Rab35 cause various diseases, including mental disorders [64].

### Neuron projection impairment

Studies have reported that many ASD risk genes are related to axonal growth [65–67]. Coordinated regulation of actin cytoskeleton is critical for proper axon growth in the growth cone during neuronal development [35]. We identified Pak3, Gap43, PC(38:0), and PC(O-42:2) involved in the JNK and MAPK/ERK pathways to regulate the actin cytoskeleton (Fig. 6c). Pak3 is a core regulator for actin cytoskeleton dynamics. In our analysis, *Pak3* was downregulated in both the mouse and human ASD subjects, indicating dysregulation of the actin cytoskeleton. A previous study showed that *Pak3*-deficient mice showed impaired long-term synaptic plasticity and learning disability [68]. JNK controls axon growth/pathfinding in the growth cone, as well as neuronal polarity. The downregulation of Gap43 inhibits axon building via the JNK pathway [69]. *Gap43*-deficient mice exhibited a subset of ASD symptoms [70]. In addition, MAPK/ERK pathway was deactivated, leading to an increase in lipid metabolism (e.g., PC(38:0) and PC(O-42:2)).

Although various studies have been reported, fully understanding the pathology of *Cntnap2*-associated ASD remains a challenge. Here, we describe core pathways directly or indirectly related to *Cntnap2*, which we found by integrating various omics study results. We incorporated multidisciplinary experimental data, allowing us to focus on the molecular functions of *Cntnap2* in ASD. We concluded that the networks related to synaptic vesicle transport, mitochondria, myelin sheath, and neuronal projections were dysregulated by *Cntnap2* mutations in ASD. Based on the identification of these central biological processes, a few genes in these pathways were found to be pivotal in the pathology of *Cntnap2* KO mice and humans with *CNTNAP2* variants. These genes should be further studied within ASD models to determine whether they are central to ASD models and patients.

## Supplementary information

Supplementary Table 1

Supplementary Table 2

Supplementary Table 3

Supplementary Table 4

Supplementary Table 5

Supplementary Table 6

Supplementary Table 7

Supplementary Table 8

Supplementary Table 9

Supplementary Table 10

Supplementary Table 11

Supplementary Table 12

Supplementary Table 13

Supplementary Information

## Data Availability

All the required data to result in the same conclusions in the paper are presented in the paper and/or the Supplementary Materials. The proteome data have been deposited in the PRIDE database (PXD031656).
