# Supplementary material for: Cntnap2-dependent molecular networks in autism spectrum disorder revealed through an integrative multi-omics analysis
Source: Mol Psychiatry. 2022 Oct 17;28(2):810–21. doi: 10.1038/s41380-022-01822-1 (PMC9908544; doi:10.1038/s41380-022-01822-1)
Supplement: Supplementary file 14 — Supplementary Information [file 41380_2022_1822_MOESM14_ESM.docx]

Supplementary Information

# *Cntnap2*-dependent molecular networks of autism spectrum disorders revealed by an integrative multi-omic analysis

**This file includes:**

- Supplementary Figures S1-S4
- Supplementary Tables S1-S13
- Details of Materials and Methods
- References

**Contents**

[Title 1](#_Toc109908120)

[Supplementary Figures 4](#_Toc109908125)

[Supplementary Fig. S1. Social behavior of Cntnap2 mice from different batches. 5](#_Toc109908121)

[Supplementary Fig. S2. Spatiotemporal mapping analysis of forebrain organoid and canonical cell type marker expression of DPal cell clusters. 7](#_Toc109908122)

[Supplementary Fig. S3. Expression of OXPHOS complex in *Cntnap2* KO mouse PFC and *CNTNAP2* mutated organoid DPal’s excitatory neuron 8](#_Toc109908123)

[Supplementary Fig. S4. CNTNAP2 related neurological disease 9](#_Toc109908124)

[Supplementary Tables 10](#_Toc109908125)

[Supplementary Table S1. Proteome profiling to identify *Cntnap2* dependent protein alteration in mPFC at PSM level. 10](#_Toc109908126)

[Supplementary Table S2. Proteome profiling to identify *Cntnap2* dependent protein alteration in mPFC at protein-coding-gene levels. 10](#_Toc109908127)

[Supplementary Table S3. Fuctional enrichment analysis results of *Cntnap2* KO mice proteome profiling. 10](#_Toc109908128)

[Supplementary Table S4. Metabolome profiling to identify *Cntnap2* dependent metabolite alteration in mPFC. 10](#_Toc109908129)

[Supplementary Table S5. Transcriptome profiling to identify ASD dependent transcriptional expression changes in human PFC. 10](#_Toc109908130)

[Supplementary Table S6. Metabolome profiling to identify ASD dependent metabolite alteration in human PFC. 10](#_Toc109908131)

[Supplementary Table S7. Lipidome profiling to identify ASD dependent metabolite alteration in human PFC - DS1. 10](#_Toc109908132)

[Supplementary Table S8. Lipidome profiling to identify ASD dependent metabolite alteration in human PFC - DS2. 10](#_Toc109908133)

[Supplementary Table S9. Integrative Gene Ontology analysis results of *Cntnap2* KO mice and ASD patient multi-omics data. 11](#_Toc109908134)

[Supplementary Table S10. Single cell transcriptome analysis of healthy subject and *CNTNAP2*-associated ASD patients-derived forebrain organoid. 11](#_Toc109908135)

[Supplementary Table S11. Cell type signatures of each cell-type of *CNTNAP2*-associated ASD patients-derived forebrain organoid. 11](#_Toc109908136)

[Supplementary Table S12. Fuctional enrichment analysis results of cell-type cluster in *CNTNAP2*-associated ASD patients-derived forebrain organoid. 11](#_Toc109908137)

[Supplementary Table S13. List of the biomolecular features in the *Cntnap2*-dependent molecular network models. 11](#_Toc109908138)

[Materials and Method Details 12](#_Toc109908139)

[Experimental model and subject details 12](#_Toc109908140)

[Behavior tests 12](#_Toc109908141)

[Three-chamber social behavior test 13](#_Toc109908142)

[Chemicals 13](#_Toc109908143)

[Proteomics 14](#_Toc109908144)

[Metabolomics 15](#_Toc109908145)

[Preprocessing of the omics data 16](#_Toc109908146)

[TMT-based quantitative proteomic data 16](#_Toc109908147)

[Targeted metabolomic data 17](#_Toc109908148)

[Bulk RNA-seq data 17](#_Toc109908149)

[Untargeted metabolomic data 17](#_Toc109908150)

[Untargeted lipidomic data 18](#_Toc109908151)

[Single-cell RNA-seq (scRNA-seq) data 18](#_Toc109908152)

[Molecular ID conversion for data integration 19](#_Toc109908153)

[Differentially expressed molecular feature analysis 19](#_Toc109908154)

[Molecular feature enrichment analysis 20](#_Toc109908155)

[Functional protein network analysis 20](#_Toc109908156)

[CNTNAP2-disease association analysis 20](#_Toc109908157)

[Reference 22](#_Toc109908158)

**Supplementary Figures**

Supplementary Fig. S1. Social behavior of *Cntnap2* mice from different batches.

Supplementary Fig. S2. Spatiotemporal mapping analysis of forebrain organoid and canonical cell type marker expression of DPal cell clusters.

Supplementary Fig. S3. Expression of OXPHOS complex in *Cntnap2* KO mouse PFC and *CNTNAP2* mutated organoid DPal’s excitatory neuron.

Supplementary Fig. S4. CNTNAP2-related neurological disease.


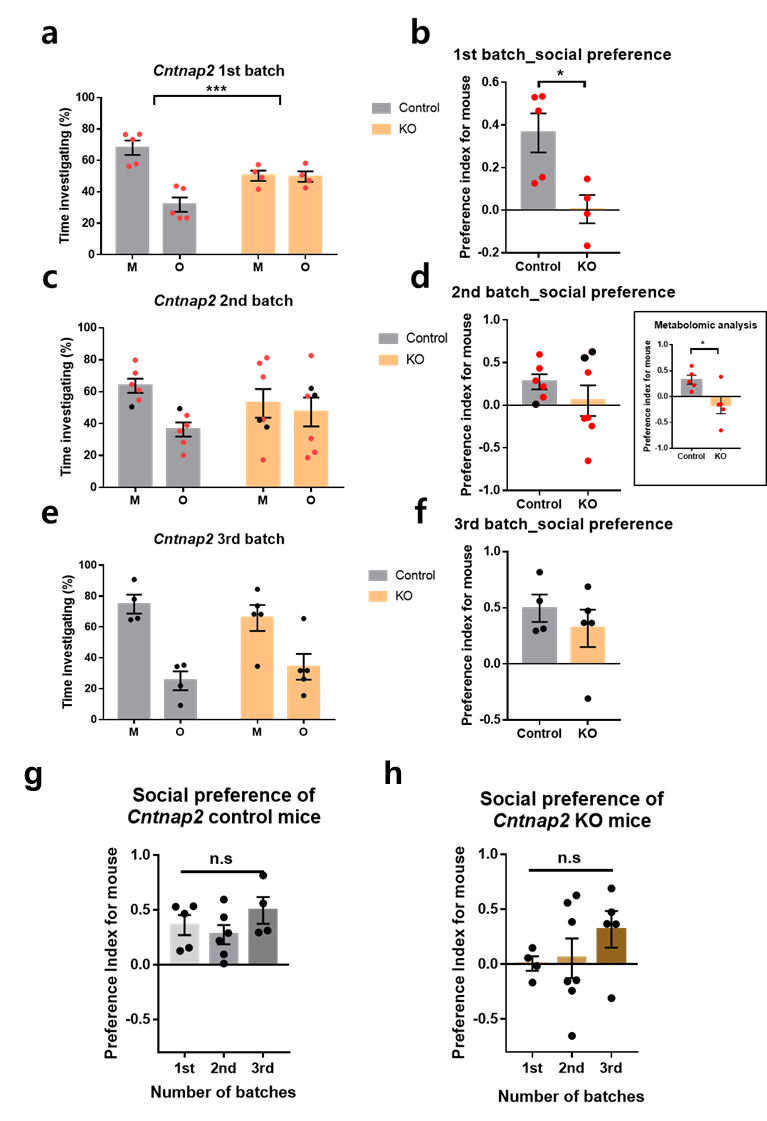


## Supplementary Fig. S1. Social behavior of *Cntnap2* mice from different batches.

(a) Bar graph showing the percentage of the time spent toward a target conspecific or an inanimate object in the first batch of control and *Cntnap2* KO mice. Two-way ANOVA ***p = 0.0008. (b) Bar graph showing preference index with significant differences between *Cntnap2* KO and control mice from the first batch. Two-tailed Welch’s t-test *p = 0.0164. These mice were used for proteomics analyses. (c) Bar graph showing the percentage of the time spent toward a target conspecific or an inanimate object in the second batch of control and *Cntnap2* KO mice. Two-way ANOVA p = 0.1541. (d) Bar graph showing preference index between *Cntnap2* KO and control mice from the second batch. Two-tailed Welch’s t-test p = 0.2999. Inset: Bar graph showing preference index with significant differences between *Cntnap2* KO and control mice used for metabolomic analyses. Two-tailed Welch’s t-test *p = 0.0392. € Bar graph showing the percentage of the time spent toward a target conspecific or an inanimate object in the third batch of control and *Cntnap2* KO mice. Two-way ANOVA p = 0.2640. (f) Bar graph showing preference index with significant differences between *Cntnap2* KO and control mice from the third batch. Two-tailed Welch’s t-test p = 0.4163. (g) *Cntnap2* control group did not show a significant difference in their distribution normality. Kolmogorov-Smirnov test, 1^st^ control, n = 5; 2^nd^ control, n = 6, 3^rd^ control, n = 4; 1^st^ control vs 2^nd^ control P = 0.5909, 2^nd^ control vs 3^rd^ control P = 0.1810, 1^st^ control vs 3^rd^ control P = 0.5635. (h) *Cntnap2* KO group did not show a significant difference in their distribution normality. Kolmogorov-Smirnov test, 1^st^ control, n = 4; 2^nd^ control, n = 7, 3^rd^ control, n = 5; 1^st^ KO vs 2^nd^ KO P = 0.6606, 2^nd^ KO vs 3^rd^ KO P = 0.7374, 1^st^ KO vs 3^rd^ KO P = 0.0794.


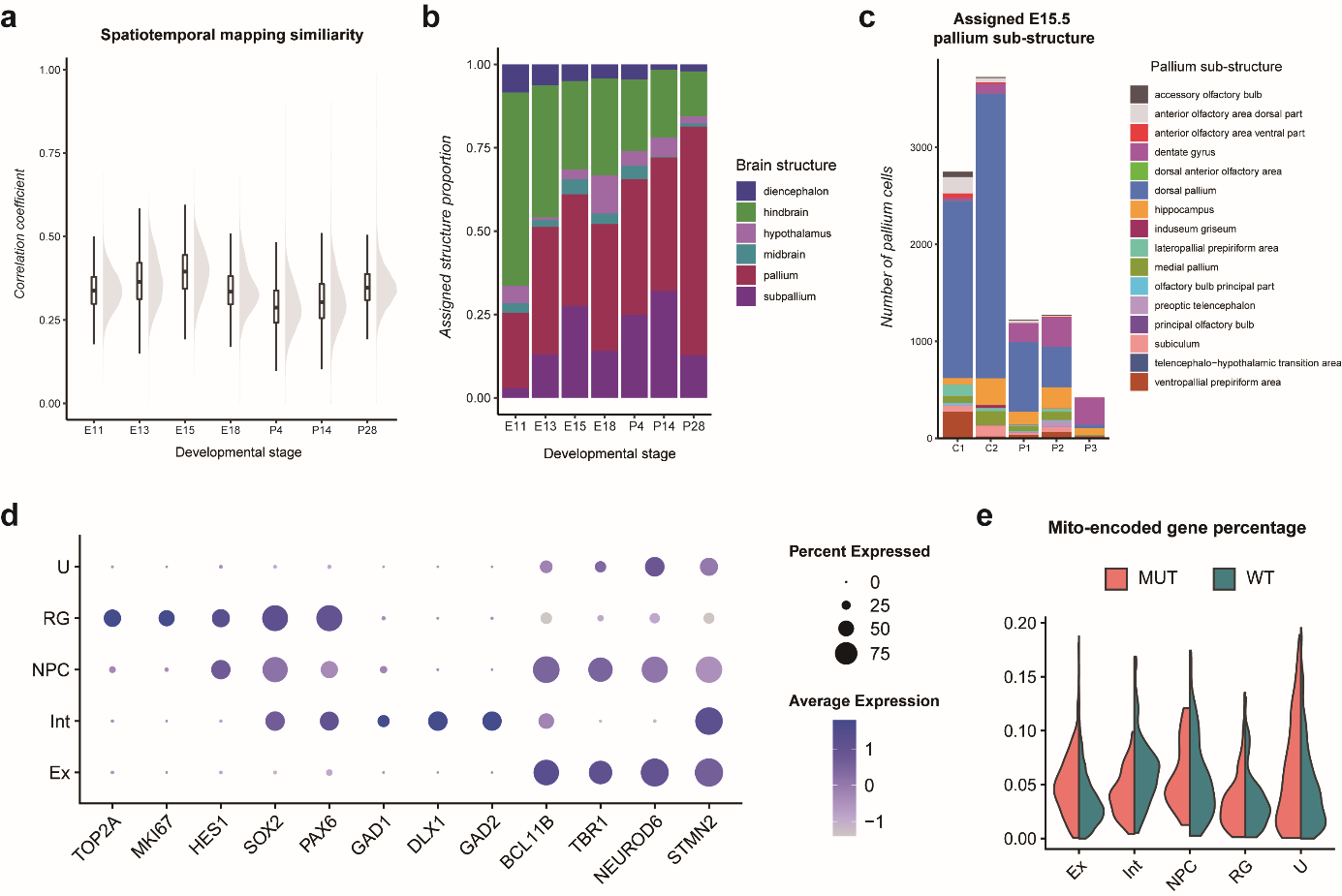


## Supplementary Fig. S2. Spatiotemporal mapping analysis of forebrain organoid and canonical cell type marker expression of DPal cell clusters.

(a) Distribution of correlation score of spatiotemporal mapping analysis. The violin plot and boxplot show the similarity scores of organoid cells mapped to highest correlated ABA voxel. (b-c) Proportion of assigned brain structures of organoid cells. Voxhunt structure annotations at level 2 (b) and level 4 (c) were shown. (d) Dot plot showing the canonical cell type marker expression per DPal cell clusters. The size of dot represents the proportions of marker gene expressing cells per each cell-type. Dot color represents the average marker gene expression per each cell-type (gray: low expression; blue: high expression). (e) Violin plot showing mitochondrial genome encoded gene expression ratio between ASD and healthy organoid per each cell-type cluster (orange: ASD patient organoid; green: healthy subject organoid).

**
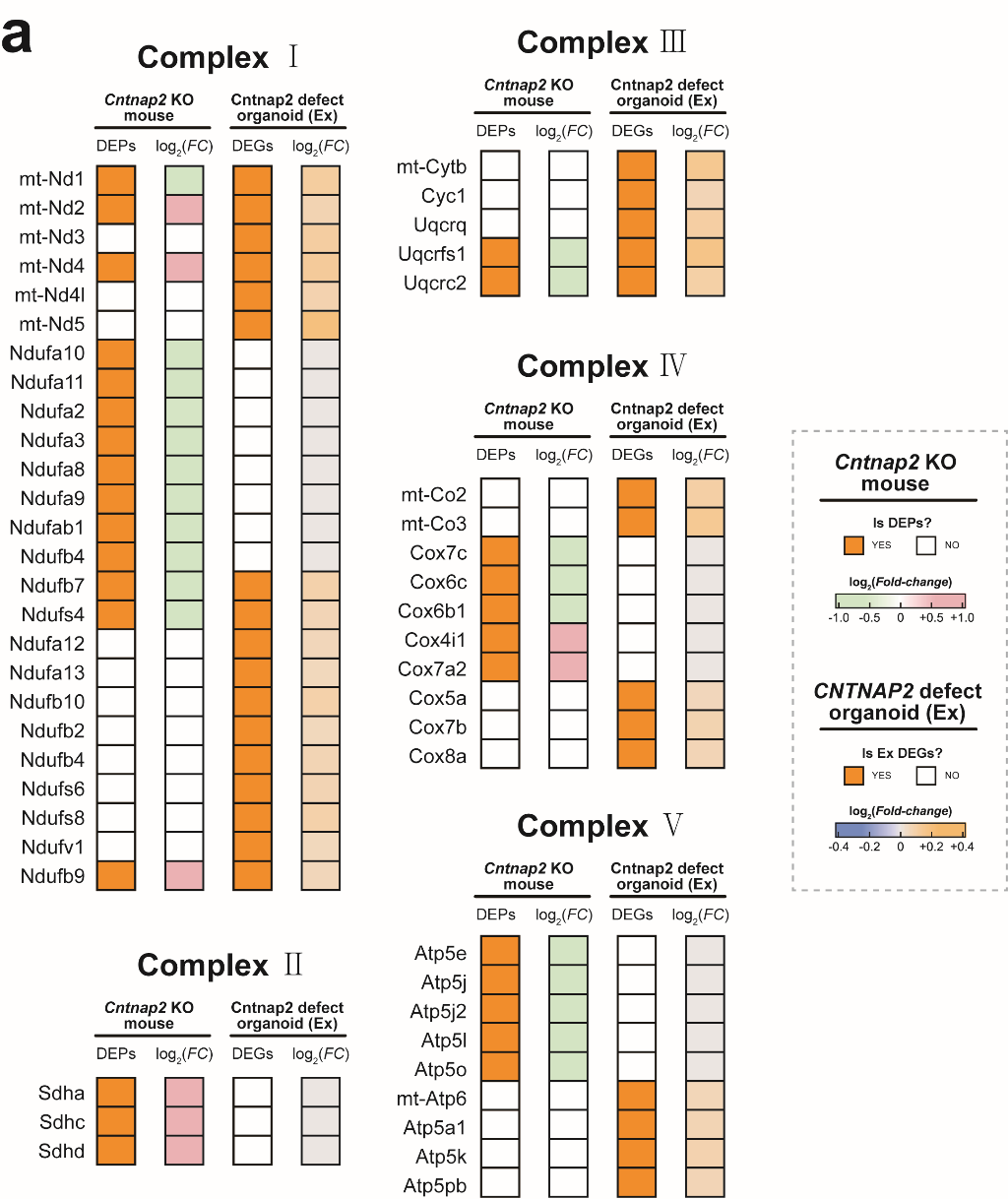
**

## Supplementary Fig. S3. Expression of OXPHOS complex in *Cntnap2* KO mouse PFC and *CNTNAP2* mutated organoid DPal’s excitatory neuron.

(a) Heatmap showing DEPs in *Cntnap2* KO mouse PFC and Ex DEG in *CNTNAP2* mutated organoid DPal cell and their corresponding expression fold-changes. DEPs in *Cntnap2* KO mPFC and Ex DEGs in ASD organoid were displayed with yellow boxs. The corresponding expression log2-fold-change in *Cntnap2* KO mPFC and Ex ASD organoid compared to control groups were also shown.

| **a** | \| **Neurological disease** \| **Genetic variants on CNTNAP2** \| **Data availability** \| \| --- \| --- \| --- \| \| Autism spectrum disorder (ASD) \| SNP^1, 2^, CNV deletion^2, 3^, CNV duplication^3, 4^, genomic rearrangement^5^ \| GSE174569^6^ (GEO; *CNTNAP2*-del ASD iPSC-organoid; scRNA-seq) \| \| Schizophrenia (SCZ) \| SNP^7, 8^, CNV deletion^9, 10^ \| GSE102838^11^ (GEO; *CNTNAP2*-del SCZ iPSC-nueron; RNA-seq) \| \| Bipolar disorder (BD) \| SNP^7^, CNV deletion^10, 12^, CNV duplication^10^ \| No data \| \| Attention deficit hyperactivity disorder (ADHD) \| CNV duplication^13^ \| No data \| \| Major depressive disorder (MDD) \| SNP^14, 15^ \| No data \| \| Alcohol dependence \| SNP^16^ \| No data \| |
| --- | --- | --- | --- | --- | --- | --- | --- | --- | --- | --- | --- | --- | --- | --- | --- | --- | --- | --- | --- | --- | --- | --- |
|  |  |
| **b** | 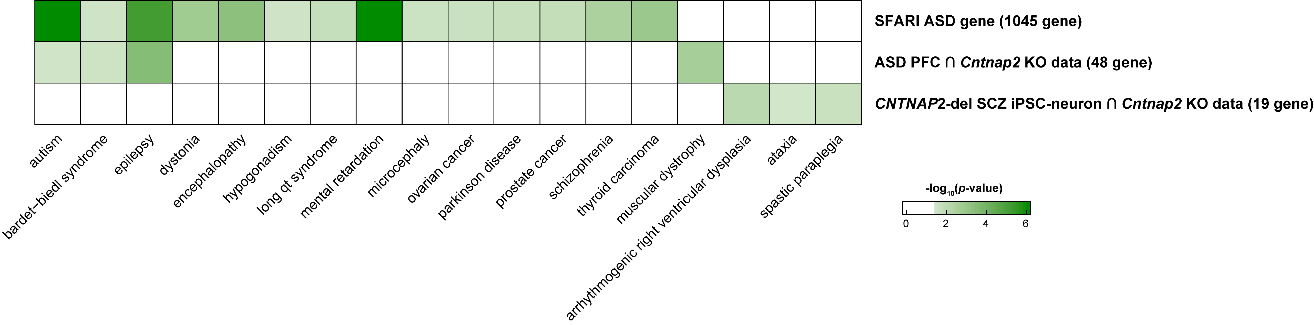 |

## Supplementary Fig. S4. CNTNAP2 related neurological disease.

(a) The table showing an overview of CNTNAP2-related genetic variants of neurological disease. (b) The heatmap showing disease association by SFARI genes, *Cntnap2*-associated ASD DEGs, and *Cntnap2*-associated SCZ DEGs. **Supplementary Tables**

## Supplementary Table S1. Proteome profiling to identify *Cntnap2*-dependent protein alteration in mPFC at PSM level.

## Supplementary Table S2. Proteome profiling to identify *Cntnap2*-dependent protein alteration in mPFC at protein-coding-gene levels.

## Supplementary Table S3. Functional enrichment analysis results of *Cntnap2* KO mice proteome profiling.

## Supplementary Table S4. Metabolome profiling to identify *Cntnap2*-dependent metabolite alteration in mPFC.

## Supplementary Table S5. Transcriptome profiling to identify ASD dependent transcriptional expression changes in human PFC.

## Supplementary Table S6. Metabolome profiling to identify ASD-dependent metabolite alteration in human PFC.

## Supplementary Table S7. Lipidome profiling to identify ASD-dependent metabolite alteration in human PFC - DS1.

## Supplementary Table S8. Lipidome profiling to identify ASD-dependent metabolite alteration in human PFC - DS2.

## Supplementary Table S9. Integrative Gene Ontology analysis results of *Cntnap2* KO mice and ASD patient multi-omics data.

## Supplementary Table S10. Single-cell transcriptome analysis of the healthy subjects and *CNTNAP2*-associated ASD patients-derived forebrain organoid.

## Supplementary Table S11. Cell type signatures of each cell-type of *CNTNAP2*-associated ASD patients-derived forebrain organoid.

## Supplementary Table S12. Functional enrichment analysis results of cell-type clusters in *CNTNAP2*-associated ASD patients-derived forebrain organoid.

## Supplementary Table S13. List of the biomolecular features in the *Cntnap2*-dependent molecular network models.

# Materials and Methods

## Experimental model and subject details

Male *Cntnap2*^-/-^ mice (Stock No: 017482) were purchased from The Jackson Laboratory (USA). *Cntnap2* ^-/-^ mice were generated by replacing the first exon with a neo gene to remove the translation initiation site^17^, which prevents the translation of *Cntnap2* protein^18-20^. These mice were bred in the animal facility at Seoul National University College of Medicine and used for breeding. In the animal facility, each male *Cntnap2*^-/-^ mouse was bred with C57BL/6 mice to produce *Cntnap2*^+/-^ mice. The *Cntnap2*^+/-^ mice from different breeding pairs were mated to produce *Cntnap2*^+/+^ (wild-type), *Cntnap2*^+/-^ (heterozygous knockout), and *Cntnap2*^-/-^ (homozygous knockout) mice. Both *Cntnap2*^+/+^ and *Cntnap2*^+/-^ mice were used as control groups in behavioral tests with *Cntnap2*^-/-^ mice, but only *Cntnap2*^+/+^ mice were used as the controls in the proteomic and metabolomic analyses. Three batches of mice were used in this study. The first batch of mice was used for the proteomic, and behavioral analyses and the second batch was used for the metabolomic and behavioral analyses, while the third batch was used only for behavioral analyses.

Three weeks after birth, each mouse was weaned from the dam. Genotyping of the *Cntnap2* variant mice was carried out according to the information provided by the Jackson Laboratory. After genotyping, the mice were raised with their littermates until they were 8 weeks old, and then they were used for the behavior tests of interest. All mice were housed in a fixed 12-hour light-dark cycle (lights on 8:00-20:00; lights off 20:00 until the next day at 8:00). Food and water were provided *ad libitum*.

## Behavior tests

All behavior tests were performed as previously described ^21^. Behavior tests were carried out in a soundproof chamber with dim light illumination during the lights-on cycle. Subjects order in the behavior test was randomly chosen. All mouse behavior was recorded during the behavior tests, and the experimenters were blinded to the experimental conditions. Both the subject and target mice were fully habituated to handling by a human before any behavior test. Mouse behavior was monitored using automated mouse behavior-tracking software (EthoVision XT 11.5, Noldus, Netherlands), and manual scoring was performed to determinemouse social interaction time.

Three batches of *Cntnap2* mice social behavior (1^st^: sample for proteomics; 2^nd^: sample for metabolomics; 3^rd^: sample for behavior test re-verification) was checked using a three-chamber social preference test. The method for a three-chamber social preference test is described in the following subsection. Interaction time between an inanimate object or target conspecific for each batch was used to compute the social preference and evaluated based on a two-way ANOVA test (**Supplementary Fig. S1a**, **S1c**, and **S1e**). The preference index of an individual batch was tested using a two-tailed Welch’s t-test (**Supplementary Fig**. **S1b**, **S1d**, and **S1f**). The normality of a preference index between groups was tested by using Kolmogorov-Smirnov normality tests (**Supplementary Fig. S1g-h**).

### Three-chamber social behavior test

The three-chamber social behavior test was performed as previously described^21^. It consisted of two consecutive sessions: (1) habituation and (2) the social preference test. In the habituation session, a subject mouse was placed in the behavior apparatus for 10 min with the chamber doors open. After the habituation session, the social preference test was performed. While the subject mouse was in the center chamber, a target mouse was placed in a wire container in either the left or right chamber. The location of the target mouse was counterbalanced; that is, an inanimate object (a mouse-shaped plastic toy) was placed in the identical wire cage on the opposite side chamber. The subject mouse was allowed to move freely for 10 min after placement of the target mouse. Interaction times with conspecifics were manually scored by researchers who were blinded to the mouse genotypes. To normalize the variation in the exploration time of each subject mouse, all data are represented by the percentage of the time spent investigating one side with respect to the total time spent investigating both sides. The preference index (PI) was calculated by the following equations (ETM, exploration time of a mouse; ETO, exploration time of an object).

$$Preference index = \frac{{ET}_{M}-{ET}_{0}}{{ET}_{M}+{ET}_{0}}$$

## Chemicals

Ammonium acetate (practical grade [p.a.]), formic acid (liquid chromatography with tandem mass spectroscopy [LC–MS] grade), phosphate-buffered saline (PBS, p.a. grade), pyridine (LC–MS grade), and phenylisothiocyanate (sequencing grade) were purchased from Sigma–Aldrich (St. Louis, MO, USA). HPLC-grade acetonitrile, ethanol, methanol, and isopropanol were purchased from Merck (Darmstadt, Germany). The water was filtered through a Millipore Milli-Q system (Milford, MA, USA).

## Proteomics

The *Cntnap2*^-/-^ and control mouse mPFC tissues were pulverized. The sample was prepared following the published filter-aided sample preparation (FASP) protocol^22^ for proteomic digestion. The pulverized tissue samples were lysed in lysis buffer containing 4% SDS, 100 mM dithiothreitol (DTT) (Roche, Penzberg, Germany, 10197777001), and 100 mM Tris-HCl (Merck Millipore, 648315) (pH 7.6/8.5) in HPLC grade water and subjected to probe sonication. A bicinchoninic acid assay (BCA) was performed to determine the exact amount of protein in each sample. The integrity of the BCA result was confirmed by SDS–PAGE with 20 µg of protein, and 180 µg of each sample was used for subsequent analyses. Additional buffer triethylammonium bicarbonate buffer (TEAB, 100 mM) (Sigma-Aldrich, St. Louis, MO, USA, T7408), 8.0 M urea, and a phosphatase inhibitor (PhosSTOP, Roche, Penzberg, Germany 4906845001) were added so that the 4% SDS lysis buffer solution was diluted to contain 0.1% SDS. The disulfide bonds were reduced by incubating the samples with DTT at 60°C for 30 min. The samples were transferred to a PBS-washed Amicon ultra 10k spin column (Merck Millipore, UFC5010). The samples were washed with 40 mM ammonium bicarbonate (ABC) (Sigma-Aldrich, St. Louis, MO, USA, 285099) followed by incubation with iodoacetamide (Sigma-Aldrich, St. Louis, MO, USA, I1149) in the dark for 30 min. The samples were washed ten times with 1 mL of TEAB buffer. After changing the collection tube, proteins were digested with MS-grade trypsin (Thermo Fisher Scientific, 90058) at a protein to enzyme ratio of 50:1 for 12 hours at 37°C. The reaction was quenched by the addition of trifluoroacetic acid to lower the pH (<2.5) of the sample. Salts and contaminants were removed on a Harvard C-18 spin column. After drying the sample, the peptides were isotopically labeled using a 10-plex tandem mass tag (TMT, Thermo Fisher Scientific, 90110). The reaction proceeded for 2 hours at room temperature at an intermediate vortex speed, and the sample was spun down every 15 min. The TMT-labeled peptide samples were dried. The extent of chemical labeling by TMT and TMT-labeling efficiency were confirmed by LC–MS/MS. Once verified, the labeled samples were pooled and resuspended in 10 mM ABC buffer and then fractionated through basic reverse-phase liquid chromatography (RPLC) (C_18_ column with a 5 μm pore size, 4.6 mm × 250 mm, XBridge, Waters) into 24 fractionated peptide samples.

The 24 obtained fractions were analyzed on an LC–MS/MS consisting of an EASY-nLC 1200 (Thermo Fisher Scientific) nanoflow liquid chromatography system and an Orbitrap Fusion Lumos Tribrid ETD mass spectrometer (Thermo Fisher Scientific) equipped with an EASY-Spray ion source. The peptides were reconstituted in 0.1% formic acid and loaded onto a trap column (100 μm inner diameter, 2 cm length, 5 μm C18 particles, Acclaim PepMap100, Thermo Fisher Scientific) before being separated on an EASY-Spray HPLC column (75 μm inner diameter, 50 cm length, and 2 μm C18 particles, PepMap RSLC, Thermo Fisher Scientific) at a flow rate of 300 nL/minute. Mobile phases A and B were composed of 0.1% formic acid (FA) and 0.1% FA in 95% acetonitrile (ACN), respectively. The peptides were resolved by changing the gradient of mobile phase B as follows: 6% to 7% in 2 min, 7% to 30% in 75 min, 30% to 100% in 2 min, 100% to 100% for 2 min, 100% to 5% for 2 min and 5% to 5% for 7 min. An MS analysis was performed in data-dependent mode (DDA) with automatic switching between MS1 and MS2. The voltage for electrospray ionization (ESI) was set to 2,300 V. MS1 and MS2 scans were acquired for the precursor ions and fragmented ions, respectively. The MS1 scan range was set to 350-1800 m/z in the “Top Speed” setting with three seconds per cycle. MS1 and MS2 scans were measured at resolutions of 120,000 and 50,000, respectively, at 200 m/z. MS2 spectra were acquired by fragmenting precursor ions with high energy collision dissociation (HCD) at 35% of the normalized collision energy. The automatic gain control (AGC) for MS1 and MS2 was set to 1,000,000 and 50,000, respectively. The isolation window for precursor ions was set to 0.7 *m/z*. Ion injection times for MS1 and MS2 were set to be 50 and 86 ms, respectively. Dynamic exclusion was set to be 25 s. Precursor ions with charge states of 2-7 were included for MS2, and singly charged ions were rejected. Internal calibration was carried out using the lock mass of polydimethylcyclosiloxane ions (*m/z* 445.1200025) produced from ambient air^23-25^.

## Metabolomics

The tissue samples were weighed and homogenized in 3 volumes of 85/15 (v/v) ethanol/10 mM phosphate buffer (pH 7.5) mixture using a Precellys homogenizer according to the manufacturer’s protocol. After homogenization, the samples were centrifuged for 5 min at 5000 g at 4°C, and the supernatants were used for targeted metabolite profiling experiments. Cleared samples were analyzed with an Absolute IDQ™ p180 kit (Biocrates Life Sciences AG, Innsbruck, Austria). The sample preparation and measurements were performed according to the manufacturer’s protocols. Briefly, 10 µL of sample was added to filter inserts (which contained internal standards) and then dried and derivatized with phenylisothiocyanate (PITC) labeling reagent. Metabolites and internal standards were then extracted with elution buffer. One-half of the eluate was measured by LC–MS/MS, and the other half was determined by flow injection analysis (FIA)-MS/MS. The analyses of the amino acids (AAs) and biogenic amines (BAs) were performed on a Zorbax Eclipse XDBC18 column (3mm ×100 mm, 3.5 µm, Agilent Technologies) at a flow rate of 0.5 ml/min with a water to acetonitrile gradient solvent system with 0.2% formic acid for 0.5-5.5 min, 0-95% acetonitrile with holding for 1 min, and 95% acetonitrile and, then, 95-2% acetonitrile for 0.5 min with a 2.5 min equilibration time and finally 2% acetonitrile. The analyses of glycerophospholipids, sphingolipids, acylcarnitines, and hexoses were performed at a flow rate of 0.03 ml/min with elution solvent provided in a kit. Multiple reaction monitoring modes of the tandem mass spectrometer (API 4000, AB Sciex) in positive ESI were used for the quantification of target metabolites. However, only hexose was analyzed in negative ESI mode.

## Preprocessing of the omics data

### TMT-based quantitative proteomic data

The LC–MS/MS data were refined by post-experiment monoisotopic mass refinement (PE-MMR) for precursor mass calibration and refinement ^26^. Using the MS-GF+ search engine (v2020.07.02), the processed spectral data were searched against the mouse proteome (UniProt DB: released November 2020) with the allowance of semi-tryptic cleavage, a maximum of 2 missed cleavages, and 10 ppm of precursor mass tolerance using the target-decoy method. TMT on a lysine residue and N-terminus and a carbamidomethylated cysteine residue were used as static modifications. Oxidation to methionine was used as a variable modification. Peptide-spectrum matches (PSMs) with a false discovery rate (FDR) ≤ 0.01 were selected from the resultant search data (mzID files) and reassigned to the peptides with the highest scored PSM per MS/MS spectrum for peptide identification. For protein inference, unique peptides aligned to a single protein-coding gene in the whole-mouse genome were selected from identified peptides. The proteins having two or more unique peptides were selected as identified proteins.

We used 10-plex TMT labeling for quantitative proteomic analysis of the mPFC in 5 *Cntnap2* KO mice (126, 127N, 127C, 128N, and 128C) and 5 control mice (129N, 129C, 130N, 130C, and 131). After correcting the isotope impurity of the TMT, the intensities of reporter ions (126-131) were extracted from all MS/MS scans on the basis of a mass tolerance of 5 ppm using in-house software. The intensity matrix of 10 reporter ions was normalized using the quantile-normalization method to correct the effect of fractionated samples. The normalized intensities were assigned to the MS/MS scans that passed the PSM FDR cut-off and were then renormalized by log2 transformation and quantile normalization for further data analysis.

### Targeted metabolomic data

Using Analyst 1.5.1 software (AB Sciex), the peaks of each target analyte were detected in and integrated from MRM transition data. After the peak integration step, the concentrations of the analysts per sample were measured and evaluated using Biocrates MetIDQ software. Specifically, the concentrations of metabolites consisting of AAs and BAs were quantified precisely with a 7-point calibration curve and internal standards, while the remaining metabolites corresponding to lipids, acylcarnitine, and hexoses were calculated semi-quantitatively on the basis of the intensities of specific stable isotope standards. For data analysis, the metabolite concentrations were converted to log2 scale data. Metabolites with a concentration lower than the limit of detection (LOD) were excluded.

### Bulk RNA-seq data

Bulk RNA-seq data of the PFC samples in ASD patients and healthy subjects obtained by Xiling Liu et al.^27^ were collected from the Gene Expression Omnibus database (GSE51264 and GSE59288 datasets), and detailed information on the samples can be found in the Liu et al study ^27^. Original datasets contained the gene expression profiles of humans (including healthy and ASD patients), chimpanzee, and macaque PFC samples. The reads per kilobase per million mapped reads (RPKM) expression matrix of the original datasets was downloaded, and then, we selected 63 human PFC datasets (containing 38 healthy and 25 ASD samples) for further analysis. The RPKM values of the selected samples were transformed to log2 scale and normalized using the quantile-normalization method (**Supplementary Table S5**).

### Untargeted metabolomic data

Untargeted metabolomic data on the PFC samples of ASD patients and healthy subjects were obtained by Ilia Kurochkin et al., and detailed information on the samples can be found in this study^28^. The original datasets consisted of two different metabolome profiles (obtained in negative and positive mode) of humans (including healthy and ASD), chimpanzees, and macaque PFC. We selected PFC data on 72 human samples (40 healthy and 32 ASD samples) for further analysis. The peak intensities of selected samples were quantile-normalized (**Supplementary Table S6**).

### Untargeted lipidomic data

Untargeted lipidomic data of the PFC samples of ASD patients and healthy subjects were obtained by Qianhui Yu et al.^29^. These data contained two sets of human PFC lipidome data (DS1 and DS2) from a healthy subjects as well as patients with various neurological disorders (ASD, schizophrenia and Downs syndrome). Detailed information on the samples can be found in the Yu et al. study^29^**.** Since the present study is focused on ASD, we selected 420 samples (in DS1; 403 healthy and 17 ASD samples) and 68 samples (DS2; 35 healthy and 33 ASD samples) from each human PFC dataset. Then, we analyzed the DS1 and DS2 data separately since they were obtained through different sample preparation, MS measurement methods, and peak annotation results. The peak intensities in the DS1 dataset were normalized using log2 transformation (**Supplementary Table S7**), whereas those in the DS2 dataset were already normalized (**Supplementary Table S8**).

### Single-cell RNA-seq (scRNA-seq) data

scRNA-seq data of the forebrain organoids used in de Jong JO et al.,^6^ were collected from the GEO database (GSE174569 dataset). These scRNA-seq data were acquired from 13-week-old human-induced PSC-derived forebrain organoids of 2 healthy control subjects (C1-2; no mutation in *CNTNAP2*) and 3 ASD patients (P1-3; homozygous c.3709DelG in *CNTNAP2*). Detailed information on the samples can be found in the JO et al. study (10)**.** After we downloaded the count matrix related to these data, a spatiotemporal mapping analysis of organoid cells was performed to investigate PFC areas.

To perform the spatiotemporal mapping analysis, we utilized *in situ* hybridization (ISH) data obtained at 7 different developmental stages (E11, E13, E15, E18, P4, P14, and P28) and located in the Allen Developing Mouse Brain Atlas (ABA). For each brain region (voxel) that is hierarchically annotated by structure ontology in ABA and VoxHunt, the gene expression profiles were determined by ISH on the basis of the VoxHunt Expression Maps (doi: 10.17632/g4xg38mwcn.1). Using the VoxHunt::structure_markers method^30^, we selected the 30 most highly expressed structure marker genes in each brain structure (VoxHunt annotation level 2)at each developmental stage. By comparing the expression of the selected structure marker to that in each organoid cell, we were able to compute Pearson correlation scores of all voxels for each cell. For each developmental stage, we assigned the voxel with the highest correlation score per cell.

Based on the spatiotemporal mapping results, we selected the developmental stage with the highest median similarity to the organoid data. The cells mapped to the dorsal pallium (DPal) voxel (VoxHunt annotation level 4) were used for further analysis. DPal cells were clustered by the Leiden clustering method using Seurat ^31^**.** The clustered cells were manually validated using neocortex markers ^32^ and canonical cell type markers. The expression profiles of these cells were converted to log2 scale and normalized using the quantile-normalization method.

## Molecular ID conversion for data integration

In the case of gene IDs in bulk RNA-seq and scRNA-seq data, each human gene was mapped to its corresponding mouse homolog gene using BioMart. The target metabolites in targeted metabolomic data were manually annotated with HMDB ID, ChEBI ID, and LipidMaps ID. When we compared our metabolomic data to humans, the untargeted metabolomics and lipidomic data, human metabolites and lipids that contained unique HMDB IDs or LipidMaps IDs were selected for data integration with our target metabolite data.

## Differentially expressed molecular feature analysis

We preprocessed the expression of the molecular features of each omics dataset as previously described. The peptide intensities were considered to represent molecular features in the proteomic analysis because the intensities of the peptides were directly measured. For all the omics data, except for the scRNA-seq data, the molecular features with expression or intensity values in more than 50% of each group of interest (the *Cntnap2* KO and control groups in the TMT-labeled proteomics and targeted metabolomic analyses and the ASD patients and healthy subject samples in bulk RNA-seq, untargeted metabolomics, and untargeted lipidomic analyses) were used. In the case of the scRNA-seq data, the genes with expression values in more than 25% of ASD organoids and healthy normal organoid cells for each cell type cluster were used in a cell-type-specific differential expression analysis. We first calculated a T-statistic value on the basis of a Student's t-test and log2-median-ratio (log2-fold-change) for each comparison of the molecular features identified through each omics dataset. We then constructed an empirical null distribution of T-statistic values and of log2-fold changes by randomly permutating the molecular features in each omics dataset (1,000 iterations). Based on two empirical null distributions, we computed adjusted p values for the t-test (Pt) and median-ratio test (Pf). For metabolome and lipidome data, the Pt and Pf per metabolite or lipid were combined using Stouffer's method (Pcom).

## Molecular feature enrichment analysis

To identify cellular processes and pathways enriched by the genes or proteins selected as differentially expressed molecular features, enrichment analysis of Gene Ontology biological processes (GOBPs), GO cellular components (GOCCs), and GO molecular functions (GOMFs) and Kyoto Encyclopedia of Genes and Genomes (KEGG) pathways was conducted using DAVID software^33^. The GO terms or KEGG pathways with a *p*-value < 0.05 and a count ≥ 2 were considered to be enriched with a gene.

## Functional protein network analysis

Using STRING-DB, we collected the interactions between a member of cell-type-specific differentially expressed molecular features from both curated protein-protein interaction (PPIs) (experimentally_determined_interaction, database_annotated, homology, and gene_fusion in STRINGDB source) and potential interactions (phylogenetic_cooccurrence, coexpression, and automated_textmining in STRING-DB source). Only the interaction with more than 0.150 STRING-DB scores were used to generate the functional protein network.

To estimate the statistical significance of the hubness of network elements, we generate the 10,000 random networks that have identical node and edge numbers to the network. We next generate the degree distribution of the random networks. Based on the degree distribution, the statistical significance (*p*-value) of network elements’ degrees were computed using a one-sided test.

## CNTNAP2-disease association analysis

The neurological disease related to CNTNAP2 genetic variants was summarized based on Toma et al.^34^(**Supplementary Fig. S4a**). The Cntnap2-related omics dataset derived from various NDDs was searched against the PRIDE database and GEO database.

For the datasets with less than three samples per condition, the molecular features within the top or bottom of 5 percentile based on fold-changes were selected as the differentially expressed molecular feature. The differentially expressed molecular feature was compared with the differentially expressed molecular feature from *Cntnap2* KO mice data. To estimate the disease association for integrative analysis results, the differentially expressed molecular features with the same trend in both public data and *Cntnap2* KO mice data were used for the OMIM disease database in Enrichr^35^ (**Supplementary Fig. S4b**).

# References

1. Arking DE, Cutler DJ, Brune CW, Teslovich TM, West K, Ikeda M *et al.* A common genetic variant in the neurexin superfamily member CNTNAP2 increases familial risk of autism. *Am J Hum Genet* 2008; **82**(1)**:** 160-164.

2. Alarcon M, Abrahams BS, Stone JL, Duvall JA, Perederiy JV, Bomar JM *et al.* Linkage, association, and gene-expression analyses identify CNTNAP2 as an autism-susceptibility gene. *Am J Hum Genet* 2008; **82**(1)**:** 150-159.

3. Girirajan S, Dennis MY, Baker C, Malig M, Coe BP, Campbell CD *et al.* Refinement and discovery of new hotspots of copy-number variation associated with autism spectrum disorder. *Am J Hum Genet* 2013; **92**(2)**:** 221-237.

4. Prasad A, Merico D, Thiruvahindrapuram B, Wei J, Lionel AC, Sato D *et al.* A discovery resource of rare copy number variations in individuals with autism spectrum disorder. *G3 (Bethesda)* 2012; **2**(12)**:** 1665-1685.

5. Bakkaloglu B, O'Roak BJ, Louvi A, Gupta AR, Abelson JF, Morgan TM *et al.* Molecular cytogenetic analysis and resequencing of contactin associated protein-like 2 in autism spectrum disorders. *Am J Hum Genet* 2008; **82**(1)**:** 165-173.

6. de Jong JO, Llapashtica C, Genestine M, Strauss K, Provenzano F, Sun Y *et al.* Cortical overgrowth in a preclinical forebrain organoid model of CNTNAP2-associated autism spectrum disorder. *Nat Commun* 2021; **12**(1)**:** 4087.

7. Wang KS, Liu XF, Aragam N. A genome-wide meta-analysis identifies novel loci associated with schizophrenia and bipolar disorder. *Schizophr Res* 2010; **124**(1-3)**:** 192-199.

8. Chen X, Long F, Cai B, Chen X, Chen G. A novel relationship for schizophrenia, bipolar and major depressive disorder Part 7: A hint from chromosome 7 high density association screen. *Behav Brain Res* 2015; **293:** 241-251.

9. Friedman JI, Vrijenhoek T, Markx S, Janssen IM, van der Vliet WA, Faas BH *et al.* CNTNAP2 gene dosage variation is associated with schizophrenia and epilepsy. *Mol Psychiatry* 2008; **13**(3)**:** 261-266.

10. Malhotra D, McCarthy S, Michaelson JJ, Vacic V, Burdick KE, Yoon S *et al.* High frequencies of de novo CNVs in bipolar disorder and schizophrenia. *Neuron* 2011; **72**(6)**:** 951-963.

11. Flaherty E, Deranieh RM, Artimovich E, Lee IS, Siegel AJ, Levy DL *et al.* Patient-derived hiPSC neurons with heterozygous CNTNAP2 deletions display altered neuronal gene expression and network activity. *NPJ Schizophr* 2017; **3**(1)**:** 35.

12. Zhang D, Cheng L, Qian Y, Alliey-Rodriguez N, Kelsoe JR, Greenwood T *et al.* Singleton deletions throughout the genome increase risk of bipolar disorder. *Mol Psychiatry* 2009; **14**(4)**:** 376-380.

13. Elia J, Gai X, Xie HM, Perin JC, Geiger E, Glessner JT *et al.* Rare structural variants found in attention-deficit hyperactivity disorder are preferentially associated with neurodevelopmental genes. *Mol Psychiatry* 2010; **15**(6)**:** 637-646.

14. Ji W, Li T, Pan Y, Tao H, Ju K, Wen Z *et al.* CNTNAP2 is significantly associated with schizophrenia and major depression in the Han Chinese population. *Psychiatry Res* 2013; **207**(3)**:** 225-228.

15. Wray NR, Pergadia ML, Blackwood DH, Penninx BW, Gordon SD, Nyholt DR *et al.* Genome-wide association study of major depressive disorder: new results, meta-analysis, and lessons learned. *Mol Psychiatry* 2012; **17**(1)**:** 36-48.

16. Zhong X, Zhang H. Linkage analysis and association analysis in the presence of linkage using age at onset of COGA alcoholism data. *BMC Genet* 2005; **6 Suppl 1:** S31.

17. Poliak S, Salomon D, Elhanany H, Sabanay H, Kiernan B, Pevny L *et al.* Juxtaparanodal clustering of Shaker-like K+ channels in myelinated axons depends on Caspr2 and TAG-1. *J Cell Biol* 2003; **162**(6)**:** 1149-1160.

18. Xing X, Zhang J, Wu K, Cao B, Li X, Jiang F *et al.* Suppression of Akt-mTOR pathway rescued the social behavior in Cntnap2-deficient mice. *Scientific Reports* 2019; **9**(1)**:** 3041.

19. Bakkaloglu B, O'Roak BJ, Louvi A, Gupta AR, Abelson JF, Morgan TM *et al.* Molecular Cytogenetic Analysis and Resequencing of Contactin Associated Protein-Like 2 in Autism Spectrum Disorders. *The American Journal of Human Genetics* 2008; **82**(1)**:** 165-173.

20. Penagarikano O, Abrahams BS, Herman EI, Winden KD, Gdalyahu A, Dong H *et al.* Absence of CNTNAP2 leads to epilepsy, neuronal migration abnormalities, and core autism-related deficits. *Cell* 2011; **147**(1)**:** 235-246.

21. Park G, Ryu C, Kim S, Jeong SJ, Koo JW, Lee YS *et al.* Social isolation impairs the prefrontal-nucleus accumbens circuit subserving social recognition in mice. *Cell Rep* 2021; **35**(6)**:** 109104.

22. Wisniewski JR, Zougman A, Nagaraj N, Mann M. Universal sample preparation method for proteome analysis. *Nat Methods* 2009; **6**(5)**:** 359-362.

23. Khan SY, Ali M, Kabir F, Renuse S, Na CH, Talbot CC, Jr. *et al.* Proteome Profiling of Developing Murine Lens Through Mass Spectrometry. *Invest Ophthalmol Vis Sci* 2018; **59**(1)**:** 100-107.

24. Ramachandran KV, Fu JM, Schaffer TB, Na CH, Delannoy M, Margolis SS. Activity-Dependent Degradation of the Nascentome by the Neuronal Membrane Proteasome. *Mol Cell* 2018; **71**(1)**:** 169-177 e166.

25. Ma SX, Seo BA, Kim D, Xiong Y, Kwon SH, Brahmachari S *et al.* Complement and Coagulation Cascades are Potentially Involved in Dopaminergic Neurodegeneration in alpha-Synuclein-Based Mouse Models of Parkinson's Disease. *J Proteome Res* 2021; **20**(7)**:** 3428-3443.

26. Shin B, Jung HJ, Hyung SW, Kim H, Lee D, Lee C *et al.* Postexperiment monoisotopic mass filtering and refinement (PE-MMR) of tandem mass spectrometric data increases accuracy of peptide identification in LC/MS/MS. *Mol Cell Proteomics* 2008; **7**(6)**:** 1124-1134.

27. Liu X, Han D, Somel M, Jiang X, Hu H, Guijarro P *et al.* Disruption of an Evolutionarily Novel Synaptic Expression Pattern in Autism. *PLoS Biol* 2016; **14**(9)**:** e1002558.

28. Kurochkin I, Khrameeva E, Tkachev A, Stepanova V, Vanyushkina A, Stekolshchikova E *et al.* Metabolome signature of autism in the human prefrontal cortex. *Commun Biol* 2019; **2:** 234.

29. Yu Q, He Z, Zubkov D, Huang S, Kurochkin I, Yang X *et al.* Lipidome alterations in human prefrontal cortex during development, aging, and cognitive disorders. *Mol Psychiatry* 2020; **25**(11)**:** 2952-2969.

30. Fleck JS, Sanchis-Calleja F, He Z, Santel M, Boyle MJ, Camp JG *et al.* Resolving organoid brain region identities by mapping single-cell genomic data to reference atlases. *Cell Stem Cell* 2021; **28**(6)**:** 1148-1159 e1148.

31. Stuart T, Butler A, Hoffman P, Hafemeister C, Papalexi E, Mauck WM, 3rd *et al.* Comprehensive Integration of Single-Cell Data. *Cell* 2019; **177**(7)**:** 1888-1902 e1821.

32. Loo L, Simon JM, Xing L, McCoy ES, Niehaus JK, Guo J *et al.* Single-cell transcriptomic analysis of mouse neocortical development. *Nat Commun* 2019; **10**(1)**:** 134.

33. Huang DW, Sherman BT, Tan Q, Kir J, Liu D, Bryant D *et al.* DAVID Bioinformatics Resources: expanded annotation database and novel algorithms to better extract biology from large gene lists. *Nucleic Acids Res* 2007; **35**(Web Server issue)**:** W169-175.

34. Toma C, Pierce KD, Shaw AD, Heath A, Mitchell PB, Schofield PR *et al.* Comprehensive cross-disorder analyses of CNTNAP2 suggest it is unlikely to be a primary risk gene for psychiatric disorders. *PLoS Genet* 2018; **14**(12)**:** e1007535.

35. Kuleshov MV, Jones MR, Rouillard AD, Fernandez NF, Duan Q, Wang Z *et al.* Enrichr: a comprehensive gene set enrichment analysis web server 2016 update. *Nucleic Acids Res* 2016; **44**(W1)**:** W90-97.
